# Excessive Leaf Rolling Reduces Grain Yield by Disrupting Source–Sink Balance in Rice (*Oryza sativa* L.)

**DOI:** 10.3390/plants15121840

**Published:** 2026-06-14

**Authors:** Guohui Li, Jiahao Zhang, Shunda Qiao, Changjin Zhu, Yuhang Zhou, Ke Xu

**Affiliations:** 1 Jiangsu Key Laboratory of Crop Genetics and Physiology, Agricultural College, Yangzhou University, Yangzhou 225009, China; mz120241412@stu.yzu.edu.cn (J.Z.); mx120250779@stu.yzu.edu.cn (S.Q.); mx120220720@stu.yzu.edu.cn (C.Z.); mz120251376@stu.yzu.edu.cn (Y.Z.); 2 Jiangsu Key Laboratory of Crop Cultivation and Physiology, Agricultural College, Yangzhou University, Yangzhou 225009, China; 3 Jiangsu Co-Innovation Center for Modern Production Technology of Grain Crops, Yangzhou University, Yangzhou 225009, China; 4 Research Institute of Rice Industrial Engineering Technology, Yangzhou University, Yangzhou 225009, China

**Keywords:** rice, plant architectype, source–sink relationship, yield, structural andnon-structural carbohydrate

## Abstract

Optimizing rice (*Oryza sativa* L.) plant architectype is an important approach to coordinating the source–sink relationship and unlocking yield potential. In this study, using the large-panicle rolled-leaf variety ST-12 and the small-panicle flat-leaf variety Nipponbare, we systematically compared plant architectype traits, photosynthetic characteristics, biomass accumulation, carbohydrate accumulation and remobilization, source–sink characteristics, and yield under two nitrogen levels in field conditions to test the hypothesis that excessive leaf rolling influences the accumulation and translocation of photosynthetic products and disrupts the source–sink balance. The results showed that Nipponbare exhibited significantly higher yield than ST-12 under both nitrogen levels, attributable to its higher number of productive panicles, grain-filling percentage, and thousand-grain weight. Although ST-12 had a higher single-leaf photosynthetic rate and leaf area index, its top three leaves were excessively rolled, reducing its canopy light interception and canopy photosynthetic rate, thereby leading to significantly lower stem NSC content at heading and biomass accumulation during grain filling compared with Nipponbare. Notably, ST-12 had higher contents of cellulose, hemicellulose, and lignin in the stem at heading, directing more pre-anthesis photosynthetic products into structural carbon, while the translocation of non-structural carbohydrates to grains was not affected. Further analysis revealed that ST-12 had a lower source capacity, sugar–spikelet ratio, source–spikelet ratio, and source–sink ratio than Nipponbare, whereas its total spikelet number and sink capacity were significantly higher. Correlation analysis showed that source characteristic indices and the source–sink ratio were positively correlated with yield, grain-filling percentage, and thousand-grain weight, while sink characteristic indices were negatively correlated with these traits. In conclusion, excessive leaf rolling impairs canopy photosynthesis, leading to a large sink but weak source imbalance. For large-panicle varieties, a higher source–sink ratio, not simply larger sink size or total biomass, is the key to high yield.

## 1. Introduction

Ensuring stable rice (*Oryza sativa* L.) yield increases is critical for global food security. Among the many factors affecting yields, plant architectype is considered to be one of the key traits determining yield potential [1]. Improving plant architectype through genetic optimization of spatial structure and organ morphology is an important approach to enhancing canopy light-use efficiency, coordinating source–sink relationships, and achieving yield breakthroughs [2,3,4].

Rice plant architectype mainly includes traits such as plant height, tiller spreading angle, leaf morphology (length, width, thickness, droopiness, and degree of rolling of the top three leaves), and panicle architectype (panicle structure, size, and weight) [1,5]. These traits are interconnected and together determine the plant’s spatial distribution pattern, light interception efficiency, capacity for photosynthetic production, and the efficiency of assimilate translocation to grains. From the perspective of source–sink theory, various plant architectype traits jointly constrain the final yield formation by influencing source strength (leaf photosynthetic capacity), sink capacity (spikelet number and grain weight), and flow efficiency (transport and partitioning of photosynthates to grains) [6].

Since the Green Revolution, dwarf breeding has greatly improved harvest index and yield of rice [7]. However, rice yield growth has entered a plateau period in recent decades. Recent studies indicate that moderately increasing plant height while simultaneously enhancing stem strength is a feasible direction to break through the current yield plateau [8]. Meanwhile, key genes regulating tiller angle, leaf morphology, and panicle architectype have been cloned [9,10,11].

The rice leaf is the primary organ for photosynthesis, and its morphological characteristics directly determine light interception efficiency, canopy microclimate, and ultimate yield potential [12]. Moderate leaf rolling (leaf rolling index of 32–44%) confers significant agronomic advantages, such as maintaining leaf erectness, reducing mutual shading between upper and lower leaves, improving light distribution within the canopy, and enhancing drought tolerance by reducing the transpiration area [12,13,14,15]. Conversely, excessive leaf rolling (rolling index >50%) drastically reduces the effective photosynthetic area, whereas insufficient rolling leads to leaf droopiness; both conditions cause yield losses [12,16,17]. Furthermore, leaf rolling often exhibits trade-off effects with other important agronomic traits, such as plant height and grain shape, collectively influencing rice yields [18,19].

Optimizing the source–sink relationship is crucial to increasing rice yields. The fundamental approach to improving grain yields lies in enhancing the capacity of the source (leaves) to produce more photosynthates, enlarging the capacity of the sink (grains), and promoting efficient translocation of carbon and nitrogen assimilates from the source to the sink [20,21]. The construction of an ideal plant architectype is essentially an overall optimization of the source–sink system. A moderate increase in plant height is beneficial for enlarging biomass accumulation, but it must be matched with stem strength and canopy structure. Optimization of the tiller angle enables more rational light distribution within the population canopy, thereby improving the population source efficiency. Improvement of leaf morphology directly enhances photosynthetic capacity and optimizes canopy light interception patterns. Optimization of the panicle architectype expands sink capacity and improves grain-filling characteristics.

Our previous studies found that the large-panicle variety ST-12 did not show a yield increase compared with the small-panicle variety Nipponbare. From the perspective of yield components, this was attributed to the significantly lower grain-filling percentage, grain weight, and harvest index of ST-12 (data unpublished). Insufficient source supply and poor assimilate translocation are important factors affecting grain filling [22,23]. ST-12 exhibits leaf rolling after the elongation stage, which may reduce its photosynthetic production capacity, alter source–sink characteristics, and consequently lead to yield reductions. In view of this, we hypothesize that the leaf-rolling trait affects yield formation by influencing the accumulation and translocation of photosynthetic products and disrupting the source–sink balance. Therefore, the objectives of this study are as follows: (1) To elucidate the effects of leaf rolling on the accumulation and translocation capacity of photosynthetic products in rice and to determine whether it limits source organ supply and assimilate partitioning to grains; (2) to reveal the effects and underlying mechanisms of leaf rolling with respect to source–sink relationships and yield formation, thereby providing theoretical references for high-yield rice cultivation and breeding.

## 2. Results

### 2.1. Temperature Conditions During the Rice Growth Period

**Figure 1:**
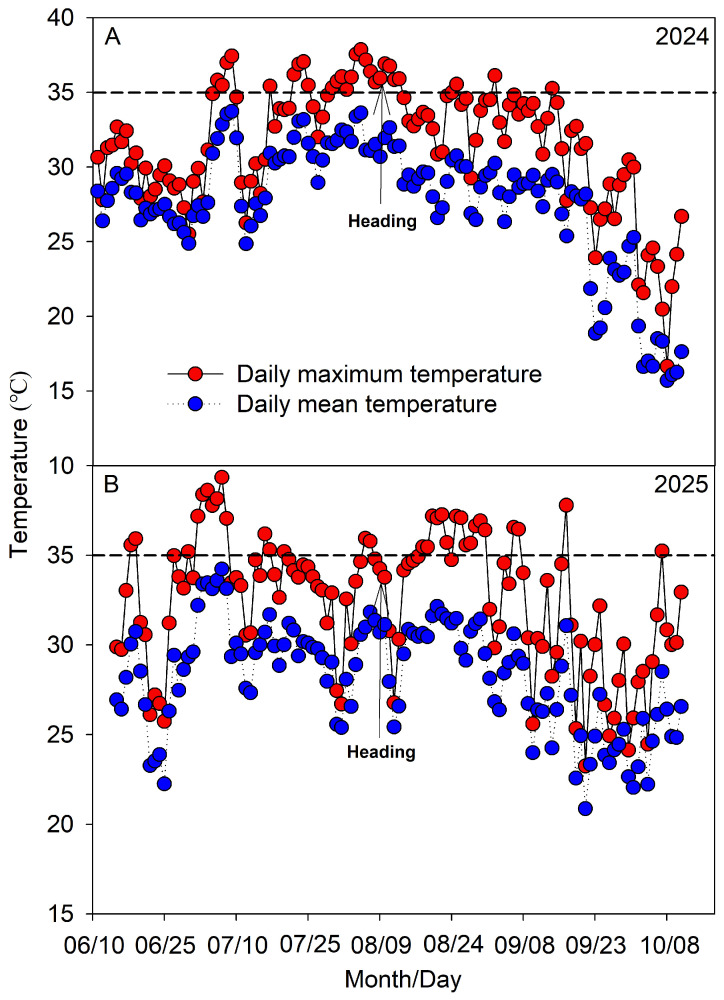
Temperature conditions during the rice growth period in 2024–2025. (**A**) Temperature in 2024; (**B**) Temperature in 2025. The dashed line represents the 35 °C threshold.

The two varieties had the same growth durations. In 2024, both varieties experienced extreme weather, with temperatures continuously above 35 °C before and for five days after heading, and the daily minimum temperature was also above 30 °C. In 2025, no high-temperature weather occurred during the heading and flowering stages of the two varieties (Figure 1). The temperature difference between the two years affected the yield performance (Table 1).

### 2.2. Rice Yield and Yield Components

Under the N0 and N240 treatments, Nipponbare exhibited higher yields than ST-12, with increases of 52.3% and 46.0% in 2024 and 15.4% and 14.4% in 2025, respectively. The yield increase was mainly attributed to its more productive panicles, grain-filling percentage, and thousand-grain weight. In 2024, under the two nitrogen levels, Nipponbare showed increases of 12.6% and 35.3% in productive panicle number, 21.0 and 28.9 percentage points in grain-filling percentage, and 31.6% and 29.3% in thousand-grain weight, respectively, compared with ST-12. In 2025, the corresponding increases were 45.4% and 47.0% in productive panicle number, 13.1 and 10.6 percentage points in grain-filling percentage, and 22.9% and 24.8% in thousand-grain weight. With increasing nitrogen level, the grain yield increased for both rice varieties. Under the N240 treatment in 2024, the yields of Nipponbare and ST-12 increased by 49.5% and 56.0%, respectively, compared with N0; in 2025, the increases were 19.3% and 20.3%, respectively. The yield increase due to nitrogen level was mainly attributed to the increase in productive panicle number (Table 1).

### 2.3. Rice Plant Architectype Traits

#### 2.3.1. Rice Plant Height, Tiller Angle, and Leaf-Rolling Rate

**Figure 2:**
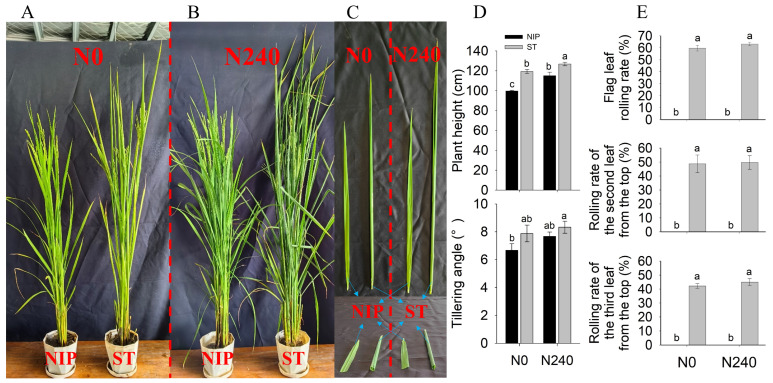
Rice plant height, tillering angle, and leaf-rolling rate under different nitrogen treatments. NIP, Nipponbare; ST, ST-12. (**A**) Plant morphology of NIP and ST under N0; (**B**) Plant morphology of NIP and ST under N240; (**C**) Flag leaf morphology of NIP and ST under N0 and N240; (**D**) Plant height and tillering angle of NIP and ST under N0 and N240; (**E**) The roilling rate of top three leaves of NIP and ST under N0 and N240. The dashed line is used to divide N0 and N240 treatments. Different letters indicate significant differences between treatments (*p* < 0.05) (Fisher’s least significant difference (LSD) test).

Under the N0 and N240 treatments, ST-12 had significantly greater plant height than Nipponbare, being 19.8% and 10.1% taller, respectively. Increasing the nitrogen level increased the plant height in both rice varieties. At the jointing and heading stages, under both nitrogen levels, ST-12 exhibited larger tiller angles than Nipponbare, with increases of 3.5% and 1.2% at the jointing stage and 16.5% and 8.6% at the heading stage, respectively; however, the differences were not significant. Increasing the nitrogen level increased the tiller angle in both varieties. Under the N240 treatment, at the jointing stage, the tiller angles of Nipponbare and ST-12 increased by 9.8% and 7.4%, respectively, compared with N0; at the heading stage, the increases were 13.4% and 5.7%, respectively, but the differences were not significant (Figure 2A,B,D).

At the heading stage and 15 days after heading, under both nitrogen levels, the top three leaves of Nipponbare showed no rolling phenotype (rolling rate of 0), whereas the rolling rates of the top three leaves of ST-12 were significantly higher than those of Nipponbare. As the growth stage progressed, the rolling rates of the top three leaves of ST-12 showed a decreasing trend, and there was no significant difference between the two nitrogen levels (Figure 2C,E).

#### 2.3.2. Rice Leaf Length, Width, and Thickness

**Figure 3:**
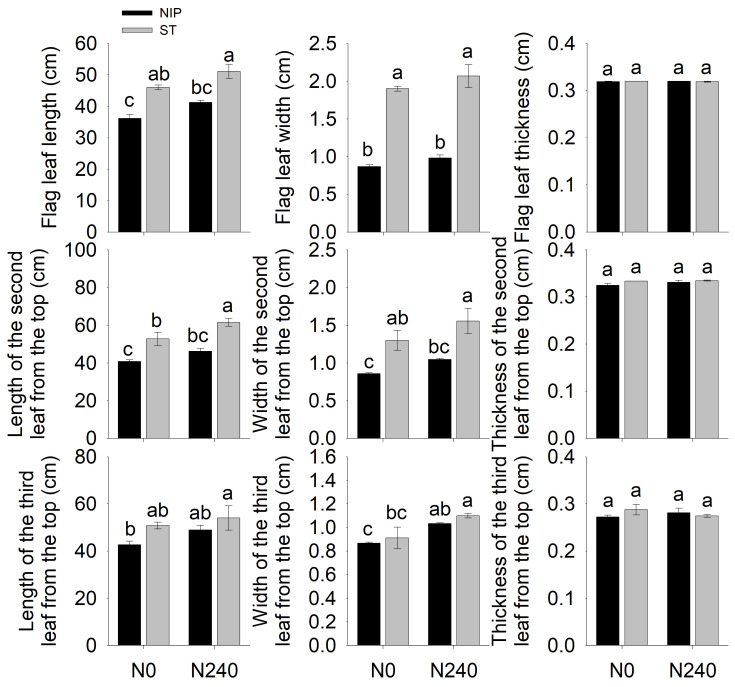
Length, width, and thickness of the top three leaves under different nitrogen treatments. NIP, Nipponbare; ST, ST-12. Different letters indicate significant differences between treatments (*p* < 0.05) (Fisher’s least significant difference (LSD) test).

At the heading stage, under the same nitrogen level, ST-12 had greater length and width of the top first, second, and third leaves than Nipponbare. Under both nitrogen levels, the leaf length and width of the top first leaf increased by 27.3% and 23.9%, and by 119.5% and 110.9%, respectively; those of the top second leaf increased by 29.1% and 33.0%, and by 51.6% and 48.6%, respectively; and those of the top third leaf increased by 19.2% and 10.2%, and by 5.1% and −3.1%, respectively. Among these, the differences in the length and width of the top first and second leaves between the two varieties were significant. Increasing the nitrogen level increased the length and width of the top three leaves but did not change their thickness; there was no difference in the thickness of the top three leaves between the two varieties under the two nitrogen treatments (Figure 3).

#### 2.3.3. Rice Leaf Droopiness

**Figure 4:**
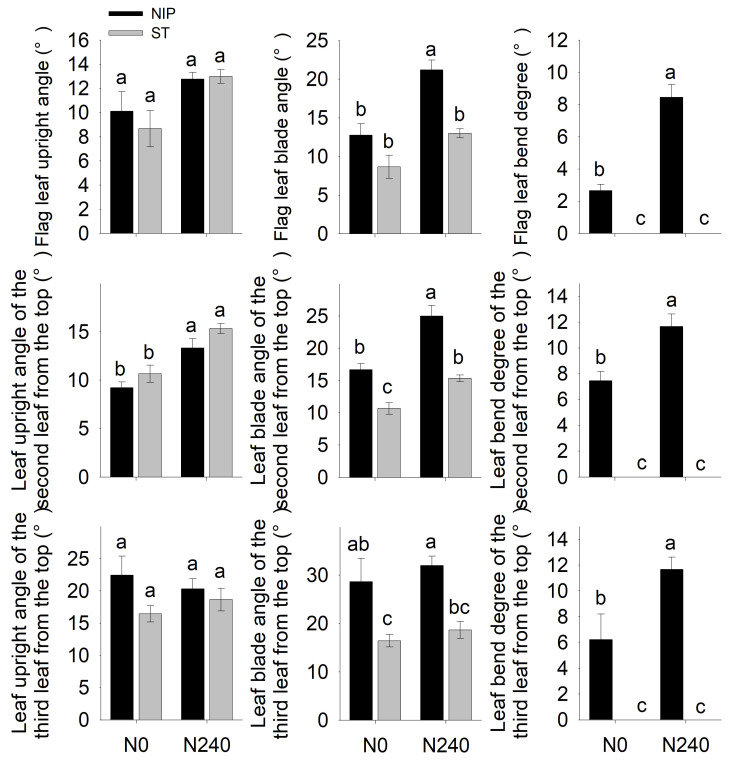
Leaf bend degree of the top three leaves under different nitrogen treatments. NIP, Nipponbare; ST, ST-12. Different letters indicate significant differences between treatments (*p* < 0.05) (Fisher’s least significant difference (LSD) test).

At the heading stage, under the same nitrogen level, except for the upright angle of the top second leaf, the upright angle and blade angle of the top first and third leaves, as well as the blade angle of the top second leaf, were higher in Nipponbare than in ST-12. Under both nitrogen levels, the top three leaves of ST-12 showed no droopiness phenotype (droopiness of 0), whereas the droopiness of the top three leaves of Nipponbare was significantly higher than that of ST-12 and increased significantly with increasing nitrogen level. Additionally, the upright angle and blade angle of the top three leaves increased in both varieties with increasing nitrogen level (except for the top third leaf of Nipponbare) (Figure 4).

#### 2.3.4. Rice Panicle Architectype

**Figure 5:**
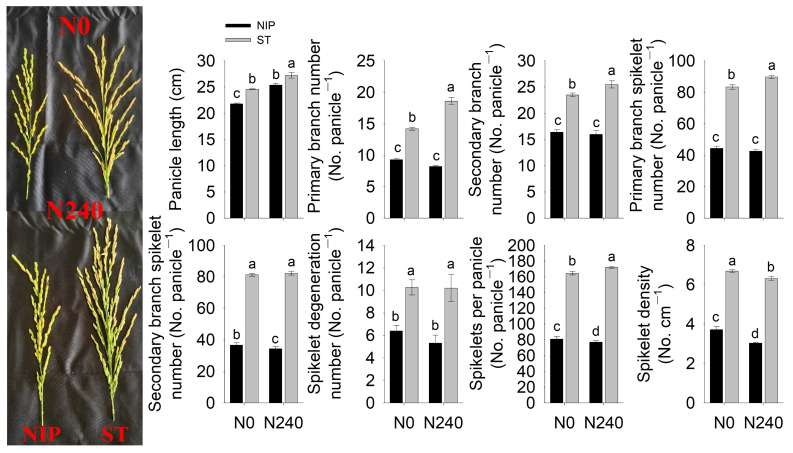
Panicle structure characteristics under different nitrogen treatments. NIP, Nipponbare; ST, ST-12. Different letters indicate significant differences between treatments (*p* < 0.05) (Fisher’s least significant difference (LSD) test).

At the heading stage, under both nitrogen levels, ST-12 showed significantly higher values for panicle architectype traits—including panicle length, number of primary branches, number of secondary branches, number of primary spikelets, number of secondary spikelets, number of degenerated spikelets, spikelet number per panicle, and spikelet density—compared with Nipponbare. Under N0 and N240, the increases were 12.8% and 7.2%, 52.7% and 126.8%, 43.3% and 59.4%, 88.0% and 110.2%, 121.8% and 139.8%, 60.4% and 92.5%, 103.3% and 123.4%, and 80.1% and 108.5%, respectively. With increasing nitrogen level, the panicle length increased significantly for both varieties, while the spikelet density decreased significantly. The number of primary branches, number of secondary branches, number of primary spikelets, and spikelet number per panicle increased significantly for ST-12 but showed no significant change for Nipponbare. The number of secondary spikelets and degenerated spikelets showed no significant change for ST-12, while the spikelet number per panicle showed opposite trends for the two varieties: increasing for ST-12 but decreasing for Nipponbare (Figure 5).

### 2.4. Rice Photosynthetic Capacity

**Figure 6:**
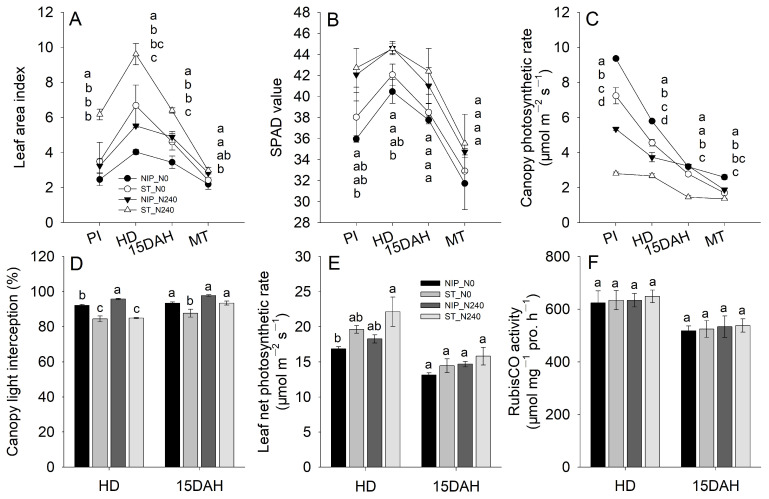
Rice photosynthetic capacity under different nitrogen treatments. NIP, Nipponbare; ST, ST-12. PI, jointing stage; HD, heading; DAH, day after heading; MT, maturity. (**A**) Leaf area index of NIP and ST under N0 and N240; (**B**) SPAD value of NIP and ST under N0 and N240; (**C**) Canopy photosynthetic rate of NIP and ST under N0 and N240; (**D**) Canopy light interception of NIP and ST under N0 and N240; (**E**) Leaf net photosynthetic rate of NIP and ST under N0 and N240; (**F**) Leaf RubisCO activity of NIP and ST under N0 and N240. Different letters indicate significant differences between treatments (*p* < 0.05) (Fisher’s least significant difference (LSD) test).

Under the same nitrogen application level, the two rice varieties showed consistent trends in leaf area index (LAI), increasing rapidly from the jointing stage to the heading stage and then gradually decreasing from the heading stage to the maturity stage. At each stage, ST-12 had a higher LAI than Nipponbare. Increasing the nitrogen level increased the LAI in both varieties (Figure 6A). Under both nitrogen levels, the SPAD values of both rice varieties showed consistent trends, increasing from the jointing stage to the heading stage and then decreasing rapidly from the heading stage to the maturity stage. Comparing the two varieties, under both nitrogen treatments, ST-12 had slightly higher SPAD values than Nipponbare, but the differences were not significant. Increasing the nitrogen level increased the leaf SPAD values (Figure 6B). Comparing the two varieties, ST-12 had higher single-leaf photosynthetic rates than Nipponbare at different stages, but the differences were not significant. Increasing the nitrogen level increased the single-leaf photosynthetic rates, and the rates decreased with advancing growth stage (Figure 6E). Comparing the two varieties, ST-12 had lower canopy photosynthetic rates than Nipponbare at different stages. Under low-nitrogen conditions, the rates were 22.7%, 21.4%, 13.0%, and 34.7% lower at the four stages, respectively; under high-nitrogen conditions, the rates were 47.7%, 28.5%, 54.9%, and 27.4% lower, respectively. Increasing the nitrogen level decreased the canopy photosynthetic rates, and the rates decreased with growth stage (Figure 6C). The canopy light interception rate of Nipponbare under two nitrogen levels was significantly higher than that of ST-12, except for the N240 treatment 15 days after heading (Figure 6D). At the two nitrogen levels, there was no difference in RubisCO activity between the two varieties of flag leaves (Figure 6F).

### 2.5. Rice Biomass

Under the two nitrogen levels, there was no significant difference in biomass between the two varieties at the jointing stage; however, from the heading stage onward, ST-12 had significantly higher biomass than Nipponbare at all stages, with the largest difference at maturity. In 2024, under the two nitrogen levels, ST-12 was 16.4% and 7.7% higher than Nipponbare, respectively, whereas in 2025, it was 17.9% and 15.1% higher than Nipponbare, respectively (Table 2).

### 2.6. Rice Carbohydrate Accumulation and Translocation

**Figure 7:**
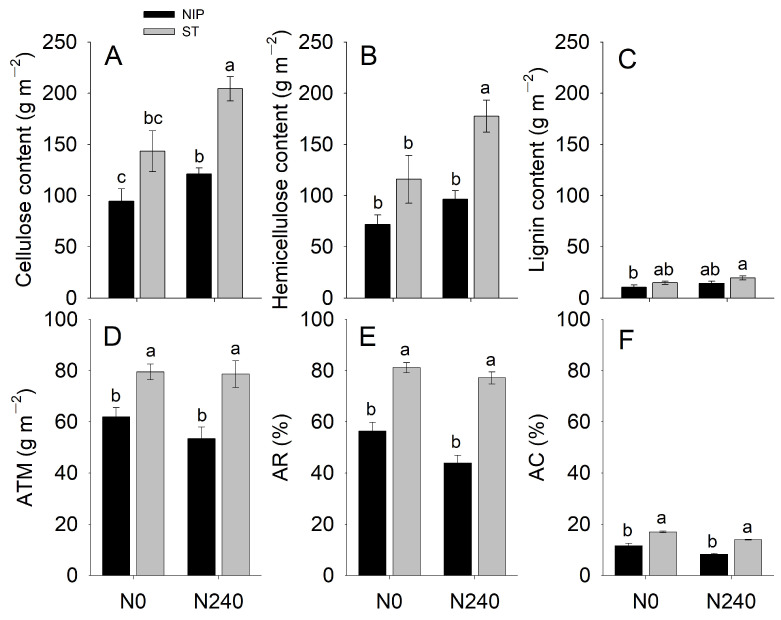
Rice carbohydrate accumulation and translocation under different nitrogen treatments. NIP, Nipponbare; ST, ST-12. (**A**) Cellulose content of NIP and ST under N0 and N240; (**B**) Hemicellulose content of NIP and ST under N0 and N240; (**C**) Lignin content of NIP and ST under N0 and N240; (**D**) Stem ATM of NIP and ST under N0 and N240; (**E**) Stem AR of NIP and ST under N0 and N240; (**F**) Stem AC of NIP and ST under N0 and N240. Different letters indicate significant differences between treatments (*p* < 0.05) (Fisher’s least significant difference (LSD) test).

Comparing the two varieties, ST-12 had higher contents of cellulose, hemicellulose, and lignin in the stem at the heading stage than Nipponbare. Under the N0 and N240 treatments, the increases were 51.6% and 68.7% for cellulose, 61.2% and 84.3% for hemicellulose, and 43.3% and 37.3% for lignin, respectively. Increasing the nitrogen level increased the contents of cellulose, hemicellulose, and lignin (Figure 7A–C). Under both the N0 and N240 treatments, ST-12 exhibited significantly higher stem ATM, AR, and AC than Nipponbare. The percentage increases in ATM were 28.4% under N0 and 47.0% under N240; for AR, the increases were 24.8 and 33.2 percentage points under N0 and N240, respectively; for AC, the increases were 5.4 and 5.7 percentage points, respectively. Across treatments, ATM, AR, and AC all decreased as the nitrogen application rate increased (Figure 7).

### 2.7. Rice Carbon Metabolism Enzyme Activities

**Figure 8:**
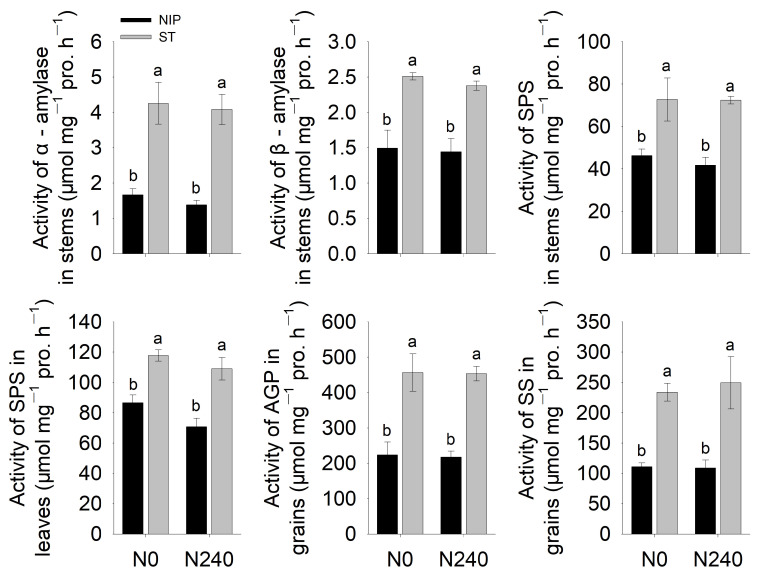
Carbon metabolism enzyme activities in rice plants under different nitrogen treatments. NIP, Nipponbare; ST, ST-12. Different letters indicate significant differences between treatments (*p* < 0.05) (Fisher’s least significant difference (LSD) test).

The stem activities of α-amylase and β-amylase were significantly higher in ST-12 than in Nipponbare. Under the N0 and N240 treatments, the α-amylase activity in ST-12 was 156.2% and 194.0% higher than that in Nipponbare, respectively, while its β-amylase activity was 68.1% and 64.8% higher, respectively. Similar trends were observed for SPS activity in stems and leaves. Under N0 and N240, the stem SPS activity in ST-12 was significantly increased by 57.4% and 73.2%, respectively, compared to Nipponbare, and the leaf SPS activity in ST-12 was significantly increased by 35.8% and 53.8%, respectively. Under the same N treatments, the grain AGP and SS activities were also significantly higher in ST-12 than in Nipponbare, with percentage increases in AGP activity reaching 120.0% and 98.4%, respectively, and those for SS activity reaching 104.3% and 144.6%, respectively. No significant changes in SS and AGP activities were observed under either N treatment (Figure 8).

### 2.8. Rice Sugar Transporter Gene Expression

**Figure 9:**
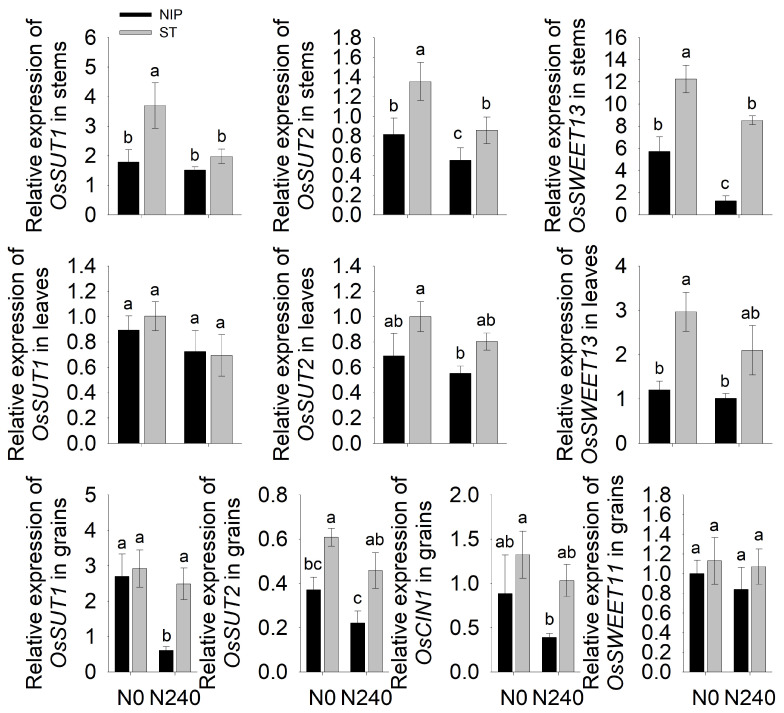
Relative expression levels of sugar transporter proteins in rice plants under different nitrogen treatments. NIP, Nipponbare; ST, ST-12. Different letters indicate significant differences between treatments (*p* < 0.05) (Fisher’s least significant difference (LSD) test).

Under the N0 and N240 treatments, the expression levels of *OsSUT1*, *OsSUT2*, and *OsSWEET13* in stems were significantly higher in ST-12 than in Nipponbare, except for *OsSUT1* under N240. The expression levels of these genes in ST-12 increased by 107.2%, 65.8%, and 114.6% under the N0 treatment and by 30.3%, 55.0%, and 577.4% under the N240 treatment, respectively, compared to those in Nipponbare. Similar results were observed in the leaves; however, no significant difference was observed in *OsSUT1* expression. The increases in *OsSUT2* and *OsSWEET13* were 45.1% and 45.4%, and 146.3% and 107.2% under N0 and N240, respectively. No significant difference was observed in *OsSUT1* expression in grains between ST-12 and Nipponbare under N0 or in *OsCIN1* and *OsSWEET11* expression under both N0 and N240. The expression levels of *OsSUT1* under N240 and of *OsSUT2* under both N treatments were significantly higher in ST-12 than in Nipponbare. An increase in the N application rate reduced the gene expression levels in stems, leaves, and grains (Figure 9).

### 2.9. Rice Source–Sink Relationship

**Figure 10:**
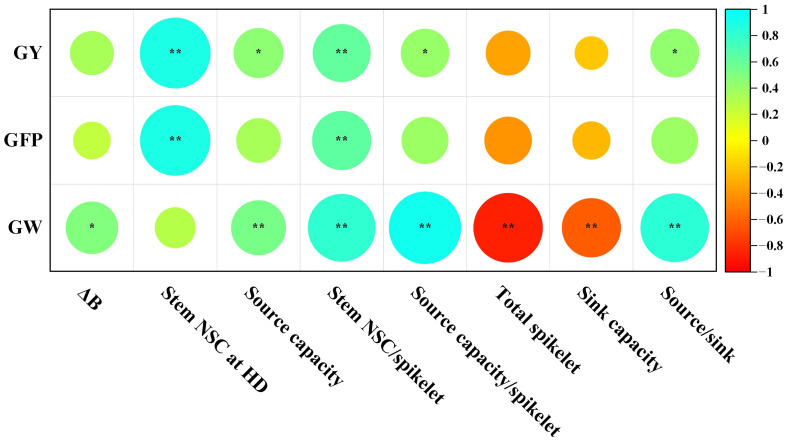
Correlations between source–sink characteristics and yield, grain-filling percentage, and thousand-grain weight (*n* = 24). GY, grain yield; GFP, grain-filling percentage; GW, grain weight. *, *p* < 0.05; **, *p* < 0.01.

Under the two nitrogen levels, for source characteristic indices, Nipponbare was significantly higher than ST-12 in both years. In 2024, under the N0 treatment, ⊿B, stem NSC content at heading, source capacity, sugar–spikelet ratio, and source–spikelet ratio increased by 20.7%, 8.7%, 103.4%, 119.9%, and 18.8%, and by 26.1%, 19.9%, 25.3%, 104.7%, and 114.4% under N240 treatment, respectively. In 2025, the increases were 14.0%, 17.8%, 12.4%, 19.5%, and 13.7% under N0 and 18.1%, 55.2%, 63.4%, 57.1%, and 63.2% under N240, respectively. For sink characteristic traits, Nipponbare was significantly lower than ST-12. In 2024, under the N0 and N240 treatments, the total spikelet number and sink capacity were reduced by 46.4% and 41.5%, and by 29.6% and 24.3%, respectively. In 2025, the reductions were 27.8% and 27.4%, and 11.3% and 9.4%, respectively. Under the two nitrogen levels, the source–sink ratio of Nipponbare was significantly higher than that of ST-12. With increasing nitrogen level, both source characteristic indices and sink characteristic indices showed increasing trends for both varieties, while the source–sink ratio showed no change (Table 3). Source characteristic traits and the source–sink ratio were positively correlated with yield, grain-filling percentage, and grain weight, whereas sink characteristic indices were negatively correlated with yield, grain-filling percentage, and grain weight (Figure 10).

## 3. Discussion

### 3.1. Leaf Rolling Compromises Canopy Photosynthesis and Carbon Allocation

Rice yield formation is a complex source–sink coordination process, and plant architectype directly influences the source–sink relationship by regulating light interception, matter production, and partitioning. In this study, although ST-12 had significantly higher biomass at the heading and maturity stages as well as greater plant height than Nipponbare, its yield was lower, indicating that simply increasing the plant height and biomass accumulation does not necessarily result in higher actual yields. Previous studies have shown that moderately increasing plant height while simultaneously enhancing stem strength can improve yields [8]; however, increased plant height was not accompanied by proportional enhancement of source capacity, leading to source–sink imbalance in ST-12, leading to intensified competition between vegetative and reproductive growth. Notably, ST-12 had a higher single-leaf photosynthetic rate and leaf SPAD values than Nipponbare, but its canopy photosynthetic rate was significantly lower than that of Nipponbare (Figure 6D). This seemingly contradictory result reveals the important regulatory effect of leaf morphology on canopy light interception. The top three leaves of Nipponbare were distinctly droopy, which favors canopy light interception and, thus, improves the canopy photosynthetic efficiency; in contrast, the leaves of ST-12 were rolled but erect, reducing canopy light interception and increasing light penetration loss, thereby resulting in a lower canopy photosynthetic rate. Moderate leaf rolling is beneficial for maintaining the upright state of leaves, promoting light energy absorption, delaying leaf aging, and increasing dry matter accumulation, while excessive leaf rolling is not conducive to yield [12,24]. Furthermore, although ST-12 had a higher leaf area index, the proportion effectively contributing to canopy photosynthesis might be lower; therefore, the ideal leaf type should aim for canopy photosynthesis rather than single-leaf photosynthesis. On the other hand, despite its larger total biomass, ST-12 had a lower biomass increment during grain filling than Nipponbare (Table 2). Dry matter production during grain filling directly determines the final grain yield, and its contribution even exceeds that of non-structural carbohydrates stored in stems and sheaths before heading. Maintaining efficient photosynthetic function during late grain filling that matches the grain-filling demand is a key link to achieving super-high yields [25,26]. Therefore, plant height improvement must be integrated with source–sink coordination and matter-partitioning efficiency. Moreover, ST-12 had significantly higher contents of cellulose, hemicellulose, and lignin in the stems than Nipponbare (Figure 7A–C), indicating that more photosynthates were used to construct structural carbon skeletons rather than being stored as non-structural carbohydrates (NSCs) or translocated to grains. This carbon partitioning strategy increased the total biomass but reduced the reserve of mobilizable carbon sources during grain filling. Consequently, although ST-12 had a large total biomass, its effective carbon proportion was low, resulting in a significantly lower source capacity, source–spikelet ratio, and sugar–spikelet ratio than Nipponbare (Table 3), ultimately limiting the improvement of grain-filling percentage and grain weight. The yield difference between years was likely due to high-temperature stress in 2024 (Figure 1). The contrasting weather conditions between 2024 (extreme heat) and 2025 (normal temperature) provide additional insight into the interaction between leaf rolling and heat stress. In 2024, both varieties experienced temperatures above 35 °C before and after heading (Figure 1), but ST-12 suffered a much greater yield reduction relative to 2025 than Nipponbare did (Table 1). First, the rolled and erect leaves of ST-12 reduce canopy transpiration area, limiting evaporative cooling and further elevating canopy temperature under heat stress [15]. Second, heat stress aggravates source limitation by accelerating leaf senescence and reducing photosynthetic capacity [25], which disproportionately affects ST-12 because its source capacity is already low. Consequently, the already compromised source–sink balance in ST-12 is exacerbated under high-temperature conditions, leading to even lower grain-filling percentage and yield. This interaction highlights the importance of considering climatic factors when evaluating leaf-rolling traits in breeding programs.

### 3.2. Source–Sink Imbalance Drives Compensatory NSC Mobilization and Limits Yield

ST-12 was significantly superior to Nipponbare in panicle length, number of branches, spikelet number, and spikelet density (Figure 5), exhibiting typical large-panicle characteristics; however, its grain-filling percentage and thousand-grain weight were significantly lower, directly reflecting a mismatch between source supply capacity and sink demand; this mismatch arises from two interlinked factors: (i) excessive leaf rolling reduces canopy photosynthesis and grain-filling biomass accumulation (Table 4), and (ii) more assimilates are diverted to structural carbon in stems, limiting mobilizable NSC reserves (Figure 7). The insufficient matter accumulation during grain filling in ST-12 resulted in a significantly lower source capacity than that of Nipponbare (Table 3). The sugar–spikelet ratio and source–spikelet ratio are key indicators for evaluating source–sink balance. In this study, the sugar–spikelet ratio and source–spikelet ratio of Nipponbare were 1.55 and 1.58 times those of ST-12 (under N240), respectively, indicating that each spikelet of Nipponbare could obtain more photosynthetic products, thereby ensuring a higher grain-filling percentage and grain weight. This result is consistent with the previous view that, for source-limited varieties, enhancing source capacity should be prioritized in order to increase yields [20]. Correlation analysis further showed that the source–sink ratio was significantly positively correlated with yield, grain-filling percentage, and thousand-grain weight; therefore, source–sink balance is of great significance for ensuring stable and high rice yields.

Notably, the apparent translocation rate (AR) and apparent contribution rate (AC) of NSCs in ST-12 were significantly higher than those in Nipponbare (Figure 7D–F). Given the lower canopy photosynthetic rate and insufficient matter accumulation during grain filling in ST-12, we propose that this high NSC translocation rate is not due to abundant source supply but rather represents a compensatory physiological response under source limitation. When leaf photosynthates cannot meet the demands of grain filling, the plant upregulates the activities of carbon-metabolizing enzymes, such as α-amylase, β-amylase, and SPS, in the stems (Figure 8) and enhances the expression of sugar transporter genes, such as *OsSUT1*, *OsSUT2*, and *OsSWEET13* (Figure 9), thereby accelerating the degradation and redistribution of stored carbohydrates in stems to sustain the carbon demand for grain filling [27,28]. This mechanism is particularly typical in large-panicle varieties with weak sources, but its cost is the excessive mobilization of structural carbon in the stem, which may weaken the lodging resistance at later stages and limit the stability of the grain-filling process.

Importantly, our study provides novel evidence that more assimilates are invested into structural carbohydrates (cellulose, hemicellulose, lignin) in stems, reducing the pool of mobilizable NSC available for grain filling in ST-12. This unfavorable carbon allocation strategy increases total biomass but lowers the effective carbon supply to grains. The resulting source limitation triggers a compensatory response, including upregulation of α-amylase, β-amylase, SPS, and sugar transporter genes, leading to the higher apparent NSC translocation rate in ST-12. However, this compensation is insufficient to rescue grain yield because the canopy photosynthetic rate remains low due to excessive leaf rolling (Figure 6C). Thus, for large-panicle varieties, simply increasing sink capacity without a synchronized enhancement of source strength, particularly canopy photosynthetic capacity and NSC reserve, will inevitably lead to a large sink but weak source imbalance and yield reduction. Our findings highlight that the yield advantage in rice does not stem from the absolute magnitude of biomass or sink capacity but rather from the coordinated balance between source and sink, specifically a high source–sink ratio.

In this study, total spikelet number and sink capacity were negatively correlated with yield, grain-filling percentage, and thousand-grain weight (Figure 10). This result was particularly prominent in ST-12, which had a significantly higher total spikelet number and sink capacity than Nipponbare but a significantly lower grain-filling percentage and grain weight. The reason for this is that the source supply capacity of ST-12 (including biomass accumulation during grain filling, stem NSC content, source capacity, etc.) could not match its excessively large sink capacity. The amount of assimilates available per spikelet (i.e., sugar–spikelet ratio and source–spikelet ratio) was insufficient, resulting in many spikelets becoming empty or shriveled due to poor grain filling. In other words, the expansion of sink capacity was not accompanied by a synchronous enhancement of source capacity; rather, it diluted the carbon supply per spikelet, leading to an imbalance of large sink but weak source. Although Nipponbare had a smaller sink capacity, it achieved higher yields by virtue of its higher source–sink ratio.

### 3.3. Implications for Breeding and Nitrogen Management

Leaf rolling, as an important plant architectype trait, has long been regarded as a double-edged sword: excessive rolling reduces the effective photosynthetic area, whereas insufficient rolling tends to cause leaf droopiness and canopy shading; both conditions result in yield losses [16,17]. However, leaf rolling is not always detrimental. A recent study showed that precise editing of the *ORL4* promoter to maintain a leaf rolling index of 32–44% not only did not reduce yields but also exhibited longer panicles, more spikelets per panicle, and higher yields [12]. Therefore, leaf rolling is a quantifiable ideal plant architectype trait. By fine-tuning source organ morphology through genome editing to maintain a slightly rolled state, the negative source–sink trade-off can be broken, achieving synergistic optimization of source morphology and sink potential. This strategy offers a highly promising technical pathway for smart design-based plant architectype customization and high-yield breeding.

In terms of breeding strategy, the source–sink ratio should be considered an important indicator for selecting high-yield varieties, rather than focusing solely on the spikelet number per panicle or biomass. In this study, although increasing nitrogen application significantly improved yield, biomass, and source characteristic values for both varieties, it did not alter the source–sink ratio between the varieties (Table 3), suggesting that the source–sink ratio differed little between nitrogen levels within each variety, indicating it is largely determined by genotype under the conditions tested. Nitrogen management can tap yield potential by enhancing the source supply and sink capacity, but it cannot fundamentally reverse the source–sink imbalance characteristic of a variety. Therefore, in high-yield cultivation, differentiated nitrogen management strategies should be adopted for different plant architectype types. For varieties with sufficient source and small sink (e.g., Nipponbare), an appropriate nitrogen increase can further expand sink capacity; for varieties with weak source and large sink (e.g., ST-12), efforts should focus on improving source capacity rather than continuing to increase the total spikelet number.

A limitation of this study is that we used two genetically distinct varieties, ST-12 and Nipponbare, that differ not only in leaf rolling but also in multiple other agronomic traits, including plant height, tillering capacity, and panicle architecture. Therefore, the observed yield differences cannot be attributed solely to leaf rolling. The use of near-isogenic lines (NILs) with similar genetic backgrounds but contrasting leaf-rolling phenotypes would be more suitable for isolating the causal effect of leaf morphology on source–sink balance. Indeed, previous studies have successfully used NILs to investigate source–sink relationships, such as the work by Akabane et al. [29] on *TGW6*, which revealed that a 1 bp deletion in tgw6 enhances source ability by increasing starch accumulation in leaf sheaths. In the context of ST-12, Miura et al. [30] developed BC2F2 progeny from a cross between Nipponbare and ST-12, which segregated for the ideal plant architecture controlled by *OsSPL14*. However, those studies focused primarily on panicle branching and tiller number, and it remains unclear whether leaf rolling co-segregated with the yield-enhancing allele in those populations. Future studies using near-isogenic lines that differ specifically in leaf rolling would help to more precisely quantify the contributions of leaf morphology versus other architectural traits to source–sink balance and yield. In addition, Nipponbare is not recognized as a high-yielding variety. In this study, the variety pair (ST-12 and Nipponbare) was selected to provide an extreme contrast in leaf morphology and panicle architecture, which is suitable for dissecting the physiological mechanisms linking leaf rolling to source–sink balance. For future agronomic validation of the practical yield penalty associated with excessive leaf rolling under regional production conditions, an additional control should be a locally adapted high-yielding Japonica variety. Despite these limitations, the present study provides novel mechanistic insights into how excessive leaf rolling redirects carbon allocation and compromises source–sink balance. Hypotheses now require causal validation using NILs and assessment of practical relevance using locally adapted high-yield controls.

## 4. Materials and Methods

### 4.1. Rice Varieties and Growth Conditions

This experiment was conducted during the 2024–2025 rice growing seasons (May to November) at the Guangling Experimental Station of Yangzhou University (Chenxing Village, Shatou Town, Yangzhou City, Jiangsu Province, 119°32′ E, 32°18′ N). The soil at the experimental site (top 0–20 cm layer) was sandy loam. The basic physical and chemical properties of the soil over the two years were as follows: pH 8.1 and 7.9; total nitrogen content 2.9 and 3.0 g kg^−1^; organic matter content 30.1 and 33.0 g kg^−1^; available phosphorus content 11.7 and 12.8 mg kg^−1^; and available potassium content 120.3 and 119.8 mg kg^−1^, respectively. The experimental site is located in a subtropical warm monsoon climate zone. Meteorological data for the two years, as shown in Figure 1, were collected by a small weather station installed near the experimental field (Onset, MA, USA). Two rice varieties (Nipponbare and ST-12) were used as materials. The ST-12, a Japonica rice variety carrying the quantitative trait locus *WFP* (*WEALTHY FARMER’S PANICLE*) encodes OsSPL14 that promotes primary branching and larger panicles, was obtained from the stocked rice collections of Togo Field and Nagoya University [30].

This experiment was conducted under field conditions with two nitrogen application levels: N0 (0 kg N ha^−1^) and N240 (240 kg N ha^−1^). These two nitrogen levels were selected to create contrasting N conditions, zero N to simulate severe source limitation and 240 kg N ha^−1^ to represent the standard high-yield N application rate in the Yangtze River Delta region, allowing us to examine the interaction between N availability and leaf rolling on source–sink balance. A split-plot design was used, with nitrogen level as the main plot and variety as the subplot. Each plot measured 4 m × 5 m = 20 m^2^, with three replications. In 2024 and 2025, seeds were sown on 20 May and 21 May, respectively, and seedlings were transplanted on 13 June and 14 June, respectively. The seedling age was 24 days, and the transplanting density was 12 cm × 30 cm, with four seedlings per hill. Nitrogen fertilizer was applied as the basal fertilizer/tillering fertilizer/panicle fertilizer = 4:2:4, applied one day before transplanting, 7 days after transplanting, and at the jointing stage (PI, approximately 40 days after transplanting), respectively. Phosphorus fertilizer (calcium superphosphate) was applied at 1000 kg ha^−1^, and potassium fertilizer (potassium chloride) was applied at 200 kg ha^−1^. All phosphorus fertilizer was applied as basal fertilizer, while half of the potassium fertilizer was applied as basal and the other half as panicle fertilizer. Alternate wetting and drying irrigation was practiced throughout the growing period, with water drainage one week before harvest. Diseases, insects, and weeds were controlled according to high-yield management practices.

### 4.2. Determination of Plant Architectype

Determination of Plant Height and Leaf Area: Plant height was measured at the heading stage on ten consecutive rice plants per plot, and the average value was calculated. Leaf area was measured at the jointing stage, the heading stage (80% heading), 15 days after heading, and the maturity stage. Green leaf area was measured using an LI-3100 area meter (LI-COR Inc., Lincoln, NE, USA), and the leaf area index (LAI, m^2^ m^−2^, leaf area per unit ground area) was calculated. Plants were separated into leaves, stems, and panicles (at the heading and post-heading stages); the plant parts were then placed in an oven, killed at 105 °C for 30 min, and subsequently dried at 80 °C to constant weight for dry weight determination.

Determination of Tiller Angle: The tiller angle was measured at the jointing stage and heading stage. The angle between the main stem and tillers was measured using a protractor. Three plants per plot were repeatedly measured, and the average value was calculated.

Determination of Leaf Morphology: At the heading stage, the length, width, thickness, droopiness, and rolling rate of the top three leaves were measured. For each plot, ten main stems of rice were sampled. Leaf length was measured from the pulvinus to the tip, and leaf width was measured at the widest part of the middle of the leaf; both leaf length and width were measured using a ruler. Leaf thickness was measured at the middle of the leaf using a digital micrometer. The angle between the leaf base and the stem (leaf upright angle) and the angle between the line connecting the leaf tip to the pulvinus and the stem (leaf blade angle) were measured using a protractor, and the leaf bend degree (leaf blade angle–leaf upright angle) was calculated [31]. The leaf width under fully expanded and natural rolled conditions was measured using a ruler, and the leaf-rolling rate was calculated as ([expanded width–rolled width]/expanded width × 100) [32]. The measurement was performed in the field on intact, attached leaves at the heading stage and 15 days after heading. Ten representative leaves per plot were measured.

Determination of Panicle Architectype: At the full heading stage, 10 main stem panicles were sampled from each plot, and the panicle length, number of primary branches, number of secondary branches, number of spikelets on primary branches, number of spikelets on secondary branches, number of degenerated spikelets, total spikelet number per panicle, and spikelet density were measured.

### 4.3. Determination of Plant Photosynthetic Capacity

Determination of Leaf SPAD Value: At the jointing stage, the heading stage, 15 days after heading, and the maturity stage, the SPAD value of the flag leaf was measured using a SPAD-502 chlorophyll meter (Konica Minolta, Inc., Osaka, Japan). Measurements were taken at the upper, middle, and lower parts of the flag leaf, and the average value was calculated as the SPAD value of that leaf. Ten leaves per plot were measured, and the average value was taken as the SPAD value for that plot.

Determination of Single-Leaf Net Photosynthetic Rate: At the heading stage and 15 days after heading, the single-leaf photosynthetic rate was measured using an LI-6400XT portable photosynthesis system (LI- COR Inc., Lincoln, NE, USA). The leaf chamber temperature was set at 30 °C, light intensity was set at 1500 μmol m^−2^ s^−1^, CO_2_ concentration was set at 400 μmol mol^−1^, and flow rate was set at 500 μmol s^−1^. Before heading, the most recent fully expanded leaf was selected; after heading, the flag leaf was selected. The photosynthetic rate at the middle part of the leaf was measured between 9:00 and 12:00 on the measurement day. After the instrument stabilized, the data were recorded. Three replicate measurements were taken per plot.

Determination of Canopy Photosynthetic Rate: At the jointing stage, the heading stage, 15 days after heading, and the maturity stage, the canopy photosynthetic rate of rice in each plot was measured. A transparent acrylic box (40 cm × 40 cm × 150 cm) was used to enclose six rice plants, and the change in CO_2_ concentration inside the box over a certain period was measured to calculate the canopy photosynthetic rate [33].

### 4.4. Determination of Stem Structural and Non-Structural Carbohydrate (NSC) Contents

At the heading stage and maturity stage, plant samples were collected. Stems were killed at 105 °C for 30 min and then dried in an oven at 80 °C to constant weight. The dried samples were ground using a small grinder (FW100, Boyn Industrial Co., Ltd., Hangzhou, Zhejiang, China), and the powder was passed through a 100-mesh sieve. One portion of the powder was used to determine the contents of cellulose, hemicellulose, and lignin in the stems. The determination was completed by Shanghai Enzyme-Linked Biotechnology Co., Ltd. (Shanghai, China). Another portion of the powder was used to determine the soluble sugar and starch contents in stems and sheaths using the anthrone colorimetric method, and the following parameters were calculated [22]:

NSC content (g m^−2^) = NSC concentration × stem dry weight;

Apparent translocation amount of NSC (ATM, g m^−2^) = NSC content at heading stage − NSC content at maturity stage;

Apparent translocation rate (AR, %) = ATM/NSC content at heading stage × 100;

Apparent contribution to yield (AC, %) = ATM/yield × 100.

### 4.5. Determination of Carbon Metabolism Enzyme Activities

At 7 days after heading (7 DAH), six plants that had flowered on the same day were selected from each plot. Their flag leaves, stems, and grains were collected, immediately frozen in liquid nitrogen, and stored at −80 °C for subsequent analysis of carbon-metabolism-related enzyme activities and expression levels of sugar-transport-associated genes. The activities of key enzymes involved in carbon metabolism—including leaf sucrose phosphate synthase (SPS); stem α-amylase, β-amylase, and SPS; and grain sucrose synthase (SS) and adenosine diphosphate glucose pyrophosphorylase (AGP)—were measured by Shanghai Enzyme-Linked Biotechnology Co., Ltd., Shanghai, China.

### 4.6. Determination of Sugar Transporter Gene Expression

The relative expression levels of several sugar-transport-related genes were quantified; these included *OsSUT1*, *OsSUT2*, and *OsSWEET13* in leaves and stems, as well as *OsSUT1*, *OsSUT2*, *OsSWEET11*, and *OsCIN1* in grains. The gene-specific primers used for reverse-transcription polymerase chain reaction (RT-PCR) are listed in Table 4. Relative expression levels for each gene were calculated using the 2^−ΔΔCT^ method [34]. For each sample, three biological replicates and three technical replicates were prepared. Gene expression measurements were conducted by Sangon Biotechnology (Shanghai) Co., Ltd., Shanghai, China.

### 4.7. Determination of Yield and Its Components

At maturity, the number of panicles was continuously investigated for 50 hills in each plot, and the number of productive panicles was calculated. Based on the average number of productive panicles per treatment, two hills were sampled, threshed, and subjected to yield component analysis. Grains were separated into filled and empty grains for counting. The grains were dried in an oven at 80 °C to constant weight. The thousand-grain weight and grain-filling percentage were calculated based on the total number of grains (sum of filled and empty grains) and the number of productive panicles. An area of 5 m^2^ in the center of each plot was harvested to determine the actual yield, which was adjusted to 14.5% moisture content.

### 4.8. Determination of Source–Sink Characteristics and Source–Sink Ratio

Source characteristic indices included biomass accumulation during grain filling (⊿B, biomass at maturity–biomass at heading), stem NSC content at heading, source capacity (biomass accumulation during grain filling + stem NSC content at heading), sugar–spikelet ratio (stem NSC content at heading/total spikelet number), and source–spikelet ratio (source capacity/total spikelet number); sink characteristic indices included the total spikelet number and sink capacity (total spikelet number × grain weight); source–sink ratio = source capacity/sink capacity [35,36].

### 4.9. Statistical Analysis

Microsoft Excel 2019 was used for data organization. Analysis of variance (ANOVA) was performed using IBM SPSS Statistics (SPSS 26.0), and the least significant difference (LSD) method was employed to compare the significance of differences among means (*p* < 0.05). Graphs were plotted using Sigma Plot software (Sigma Plot 10.0, SYSTAT Software, Inc., San Jose, CA, USA). Simple linear correlation was used to calculate correlation coefficients. The trend of yield difference (Nipponbare > ST-12) was consistent across the two years. For physiological measurements, the data from both years showed qualitatively consistent patterns, so the data from the more representative year (2025, without heat stress) are presented in the figures, with 2024 data showed as Supplementary data (Figures S1–S9).

## 5. Conclusions

The results of this study indicate that, although ST-12 has a larger sink capacity, higher single-leaf photosynthetic rate, and greater biomass accumulation, excessive leaf rolling weakens its canopy photosynthetic capacity and distributes carbon toward structural components rather than to non-structural carbohydrate reserves. This leads to a lower matter accumulation during grain filling and source–sink ratio compared with Nipponbare. Although ST-12 shows compensatory upregulation of carbon-metabolizing enzymes and sugar transporters that enhance NSC remobilization, this compensation cannot overcome the negative impact of excessive leaf rolling on assimilate supply. Therefore, the yield advantage in rice does not stem from the absolute magnitude of biomass or sink capacity but rather from the coordination capacity between source and sink. For large-panicle varieties, breeding and management should prioritize strengthening source capacity by optimizing leaf morphology to maintain moderate rolling without excessive canopy shading and improving the source–sink ratio.

## Figures and Tables

**Table 1:** Rice yield and its components under different nitrogen treatments.

Treatment/Year	Variety	Panicle (×10^4^ ha^−1^)	Spikelets Per Panicle (No. Panicle^−1^)	Grain-Filling Percentage (%)	1000-Grain Weight (g)	Yield (t ha^−1^)
2024						
N0	NIP	473.15 b	79.36 b	31.38 b	25.13 a	3.29 b
	ST	420.37 b	166.93 a	10.40 c	19.10 b	2.16 c
N240	NIP	620.37 a	73.51 b	60.23 a	25.47 a	4.92 a
	ST	458.64 b	169.81 a	31.33 b	19.70 b	3.37 b
2025						
N0	NIP	515.74 b	79.13 b	80.31 a	24.88 a	5.40 ab
	ST	354.63 d	158.89 a	67.26 b	20.24 b	4.68 b
N240	NIP	626.85 a	78.41 b	81.58 a	25.14 a	6.44 a
	ST	427.78 c	158.37 a	70.98 b	20.15 b	5.63 ab
Analysis of variance						
N treatment (N)		**	**	**	**	ns
Variety (V)		**	ns	**	ns	ns
Year (Y)		ns	ns	**	ns	**
N × V		**	ns	ns	ns	ns
N × Y		**	ns	**	**	**
V × Y		ns	ns	**	ns	**
N × V × Y		ns	ns	ns	ns	ns

NIP, Nipponbare; ST, ST-12. Different letters in the same column indicate significant differences between treatments in the same year (*p* < 0.05) (Fisher’s least significant difference (LSD) test); **, significant at the 0.01 probability level; ns, not significant at the 0.05 probability level.

**Table 2:** Rice biomass at different growth stages under different nitrogen treatments.

Treatment/Year	Variety	Jointing (t ha^−1^)	Heading (t ha^−1^)	15 DAH (t ha^−1^)	Maturity (t ha^−1^)
2024					
N0	NIP	3.28 c	6.92 c	10.67 b	12.40 c
	ST	4.59 b	9.89 b	12.93 ab	14.43 b
N240	NIP	4.51 b	9.78 b	12.39 ab	16.81 a
	ST	7.36 a	12.75 a	14.74 a	18.10 a
2025					
N0	NIP	2.36 b	7.24 c	10.23 c	12.22 d
	ST	3.48 b	10.04 b	12.81 b	14.41 c
N240	NIP	5.29 a	10.38 b	12.76 b	16.25 b
	ST	7.05 a	13.25 a	15.46 a	18.71 a
Analysis of variance					
N treatment (N)		**	**	**	**
Variety (V)		**	**	**	**
Year (Y)		ns	ns	ns	ns
N × V		ns	ns	ns	ns
N × Y		ns	ns	ns	ns
V × Y		ns	ns	ns	ns
N × V × Y		ns	ns	ns	ns

NIP, Nipponbare; ST, ST-12. Different letters in the same column indicate significant differences between treatments in the same year (*p* < 0.05) (Fisher’s least significant difference (LSD) test); **, significant at the 0.01 probability level; ns, not significant at the 0.05 probability level.

**Table 3:** Source–sink relationship under different nitrogen levels.

Treatment/ Year	Variety	Source					Sink		
		⊿B (g m^−2^)	NSC Content at Heading (g m^−2^)	Source Capacity (g m^−2^)	NSC/Spikelets	Source/Spikelets	Total Spikelets (×10^4^ m^−2^)	Sink Capacity (g m^−2^)	Source/Sink
2024									
N0	NIP	547.69 ab	89.36 b	637.04 ab	2.40 a	16.82 a	3.76 b	944.56 c	0.66 a
	ST	453.89 b	82.18 b	536.06 b	1.18 b	7.65 b	7.02 a	1341.57 ab	0.39 b
N240	NIP	674.31 a	99.26 a	773.57 a	2.19 a	16.98 a	4.56 b	1162.02 bc	0.64 a
	ST	534.68 ab	82.78 b	617.46 ab	1.07 b	7.92 b	7.79 a	1534.58 a	0.41 b
2025									
N0	NIP	497.73 bc	110.07 a	607.80 bc	2.71 a	15.00 a	4.08 c	1014.04 c	0.60 a
	ST	436.62 c	97.93 a	534.55 c	1.75 bc	9.54 b	5.64 b	1143.20 bc	0.47 b
N240	NIP	642.69 a	121.56 a	764.24 a	2.48 ab	15.59 a	4.92 bc	1236.47 ab	0.62 a
	ST	545.60 ab	101.73 a	647.34 b	1.52 c	9.61 b	6.78 a	1364.85 a	0.48 b
Analysis of variance									
N treatment (N)		**	*	**	ns	ns	**	**	ns
Variety (V)		**	*	**	**	**	**	**	**
Year (Y)		ns	**	ns	**	ns	ns	ns	ns
N × V		ns	ns	ns	ns	ns	ns	ns	ns
N × Y		ns	ns	ns	ns	ns	ns	ns	ns
V × Y		ns	ns	ns	ns	*	**	*	*
N × V × Y		ns	ns	ns	ns	ns	ns	ns	ns

NIP, Nipponbare; ST, ST-12. ⊿B, biomass accumulation during grain filling (maturity biomass − heading biomass). Different letters in the same column indicate significant differences between treatments in the same year (*p* < 0.05) (Fisher’s least significant difference (LSD) test); * and **, significant at the 0.05 and 0.01 probability level, respectively; ns, not significant at the 0.05 probability level.

**Table 4:** Primers for RT-PCR.

Gene	Primer F (5′–3′)	Primer R (5′–3′)
*OsSUT1*	TCATCCCTCAGGTGGTCATCG	CTTGGAGATCTTGGGCAGCAG
*OsSUT2*	GCATCAGCTGTGCCAACCT	CTGCTTCATCACTTCCAAAGGA
*OsSWEET11*	TGGTTCTGCTACGGCCTCTT	GGTACCAGAAGTAGAGCCCCATCT
*OsSWEET13*	CTACGCGCTGATCAAGTCCAA	GGGCGTAGGCGAGGTACAT
*OsCIN1*	CGACCCTACCAA GTCTTCTCTTAG	CCCATTGTTGAAGACGTAAAGATG
*OsUbiquitin*	CTCGCCGACTACAACATCCA	TCTTGGGCTTGGTGTACGTCTT

## Data Availability

The original contributions presented in this study are included in the article. Further inquiries can be directed to the corresponding author. The data are not publicly available due to privacy restriction.

## Supplementary Materials

The following supporting information can be downloaded at: https://www.mdpi.com/article/10.3390/plants15121840/s1, Figure S1: Plant architectype of Nipponbare and ST-12 under different nitrogen treatments; Figure S2: Rice plant height, tillering angle, and leaf- rolling rate under different nitrogen treatments in 2024; Figure S3: Length, width, and thickness of the top three leaves under different nitrogen treatments in 2024; Figure S4: Leaf bend degree of the top three leaves under different nitrogen treatments in 2024; Figure S5: Panicle structure characteristics under different nitrogen treatments in 2024; Figure S6: Rice photosynthetic capacity under different nitrogen treatments in 2024; Figure S7: Rice carbohydrate accumulation and translocation under different nitrogen treatments in 2024; Figure S8: Carbon metabolism enzyme activities in rice plants under different nitrogen treatments in 2024; Figure S9: Relative expression levels of sugar transporter proteins in rice plants under different nitrogen treatments in 2024.
