# Supplementary material for: Excessive Leaf Rolling Reduces Grain Yield by Disrupting Source–Sink Balance in Rice (Oryza sativa L.)
Source: Plants (Basel). 2026 Jun 14;15(12):1840. doi: 10.3390/plants15121840 (PMC13306753; doi:10.3390/plants15121840)
Supplement: Supplementary file 1 [file plants-15-01840-s001.zip › plants-4349918-supplementary.pdf]

Supplementary data

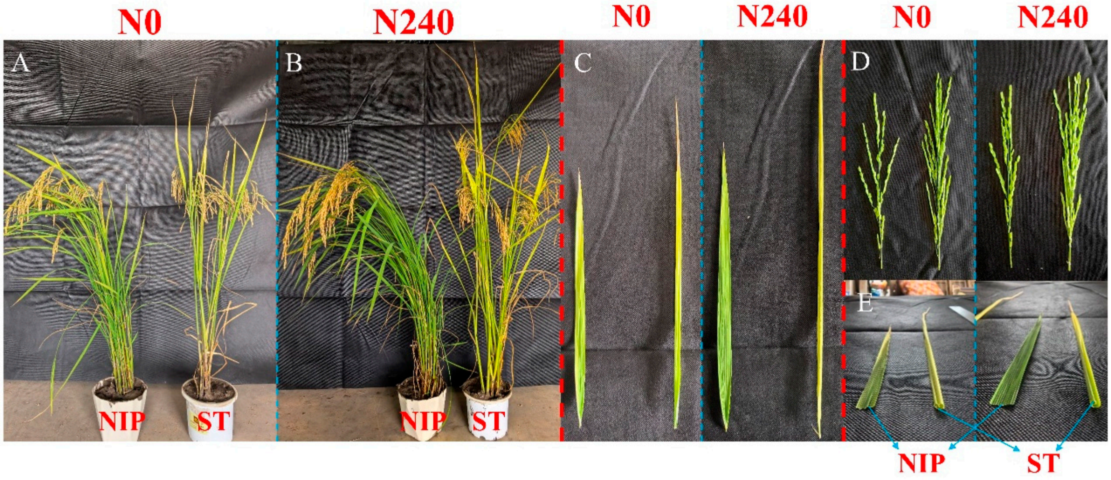

Figure S1. Plant architectype of Nipponbare and ST-12 under different nitrogen treatments. NIP, Nnipponbare; ST, ST-12.

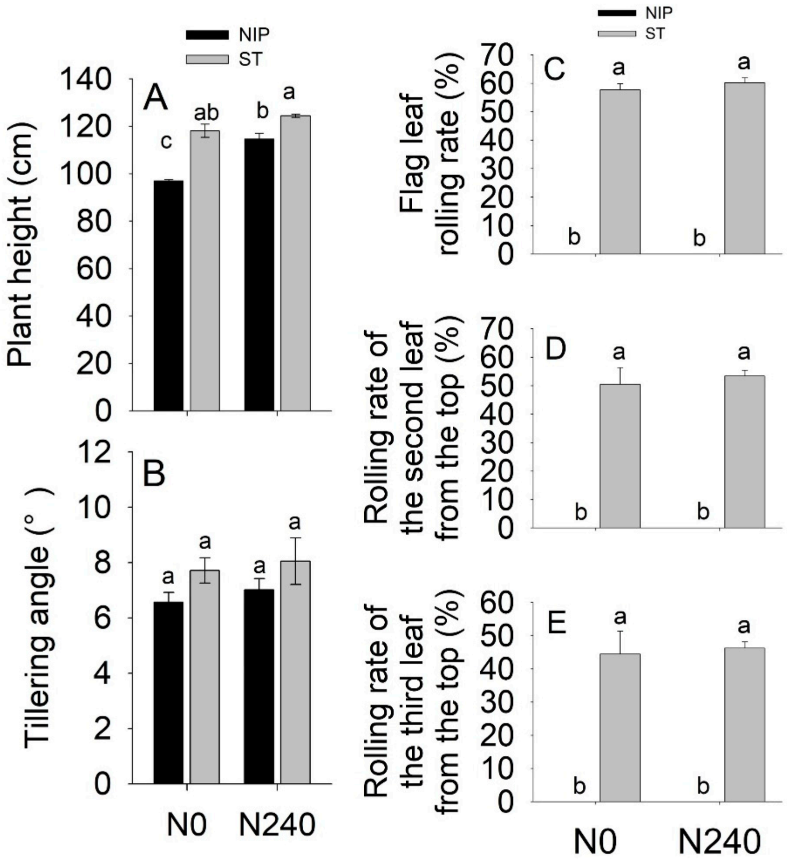

Figure S2. Rice plant height, tiller angle, and leaf- rolling rate under different nitrogen treatments in 2024. NIP, Nipponbare; ST, ST-12. Different letters indicate significant differences between treatments ($p < 0.05$) (Fisher's least signiffcant difference (LSD) test).

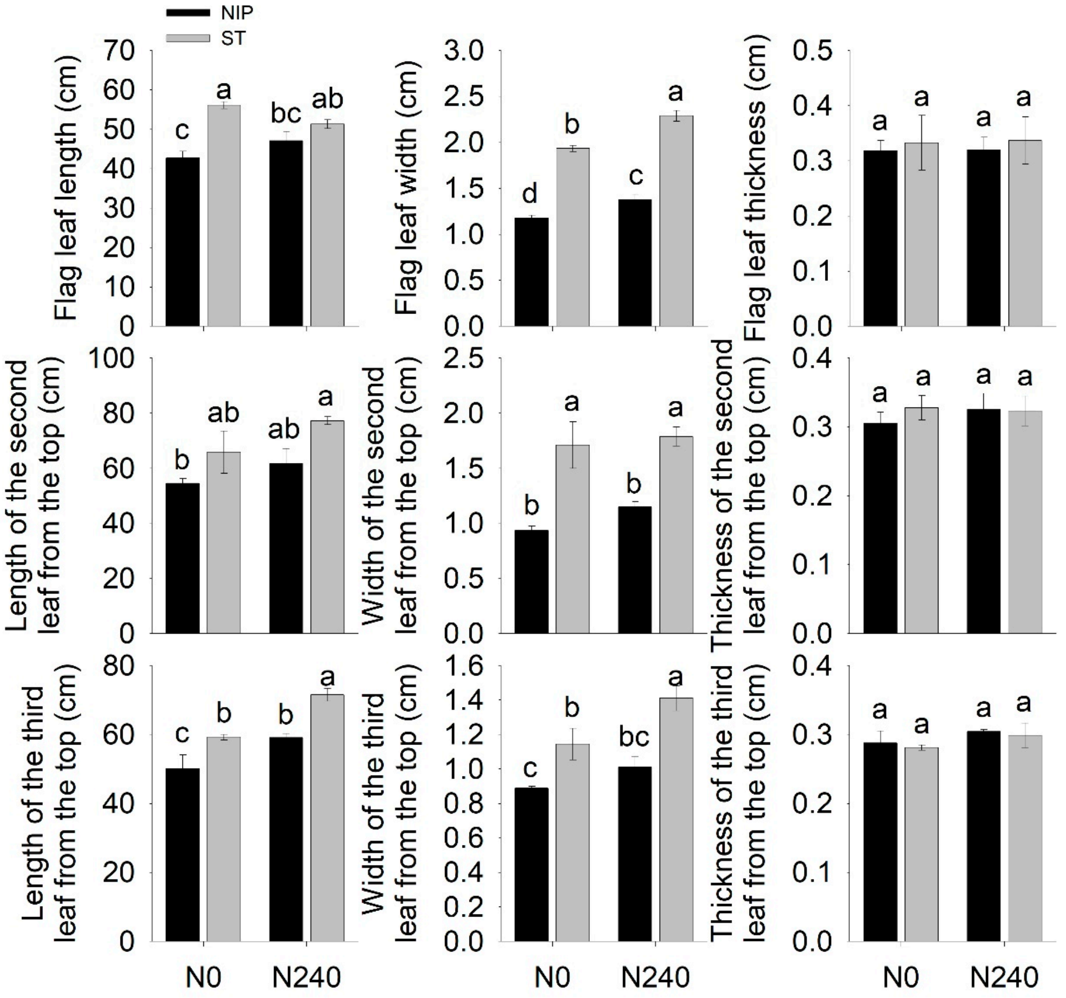

Figure S3. Length, width, and thickness of the top three leaves under different nitrogen treatments in 2024. NIP, Nipponbare; ST, ST-12. Different letters indicate significant differences between treatments ($p < 0.05$) (Fisher's least signiffcant difference (LSD) test).

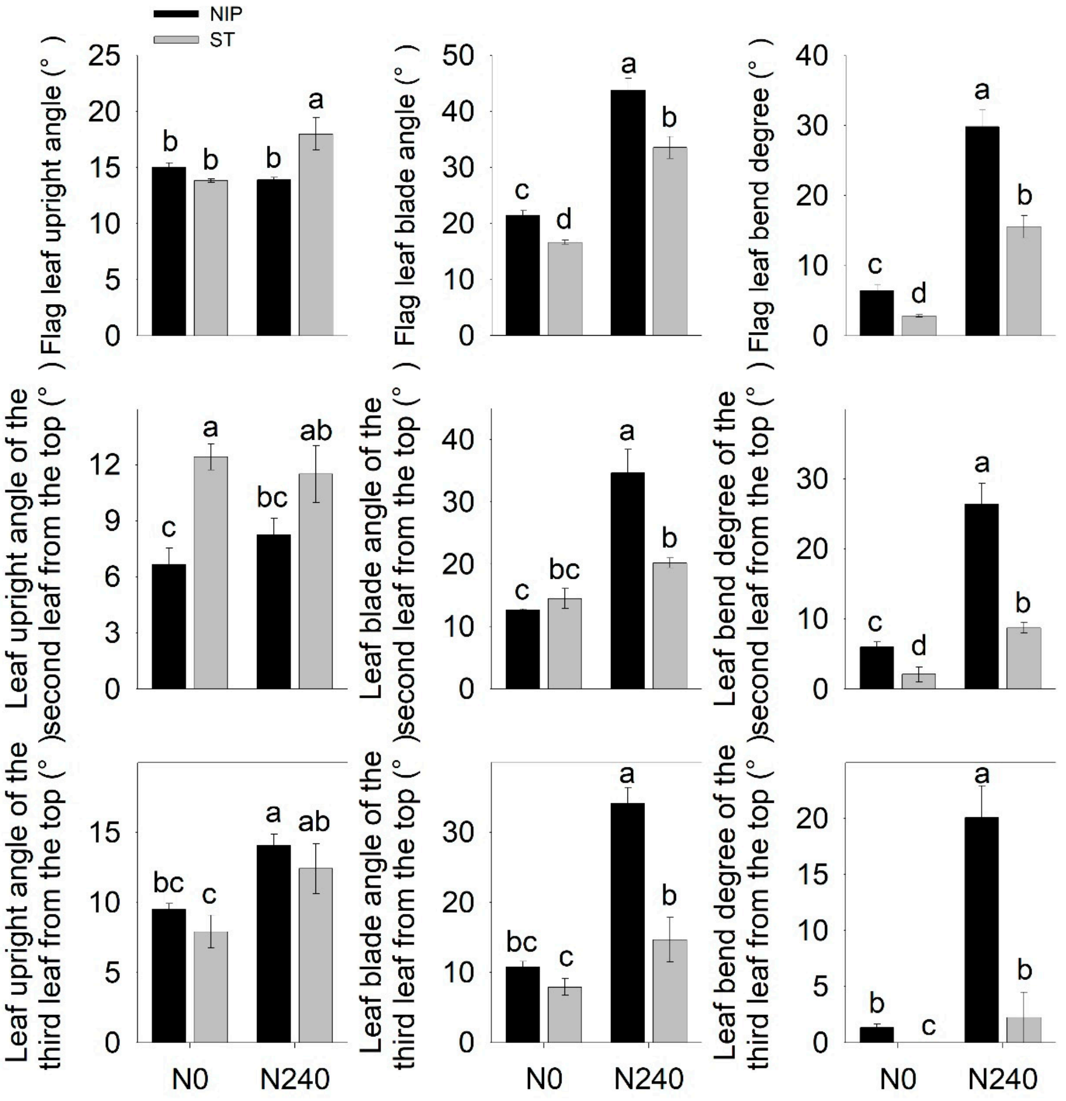

Figure S4. Leaf bend degree of the top three leaves under different nitrogen treatments in 2024. NIP, Nipponbare; ST, ST-12. Different letters indicate significant differences be-tween treatments ($p < 0.05$) (Fisher's least signiffcant difference (LSD) test).

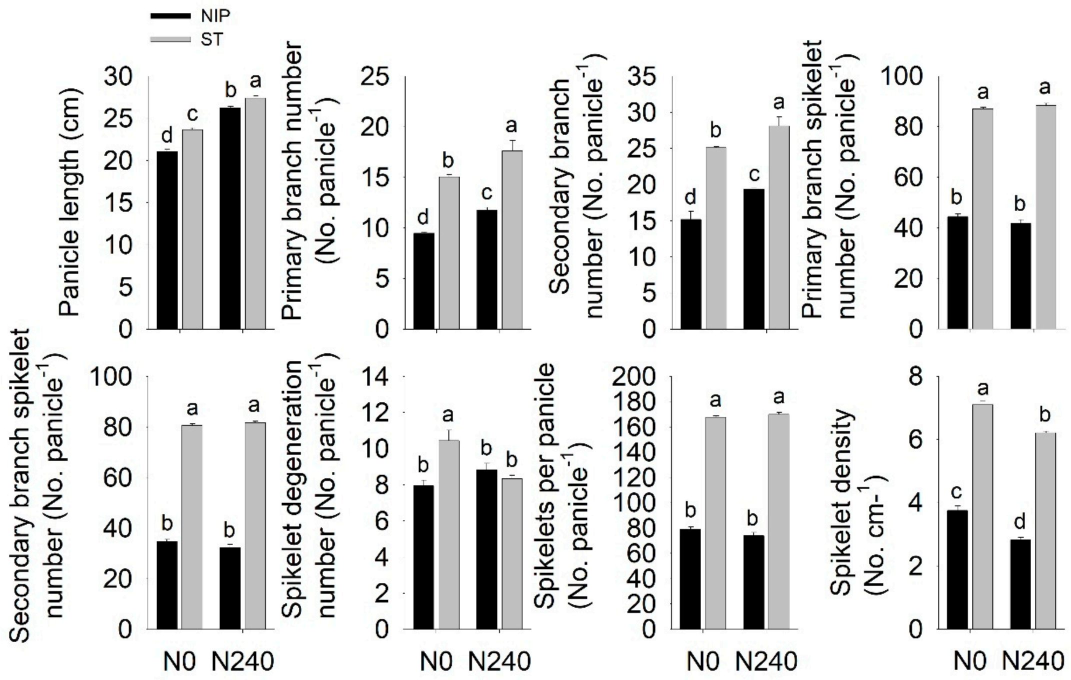

Figure S5. Panicle structure characteristics under different nitrogen treatments in 2024. NIP, Nipponbare; ST, ST-12. Different letters indicate significant differences between treatments (p < 0.05) (Fisher's least signiffcant difference (LSD) test).

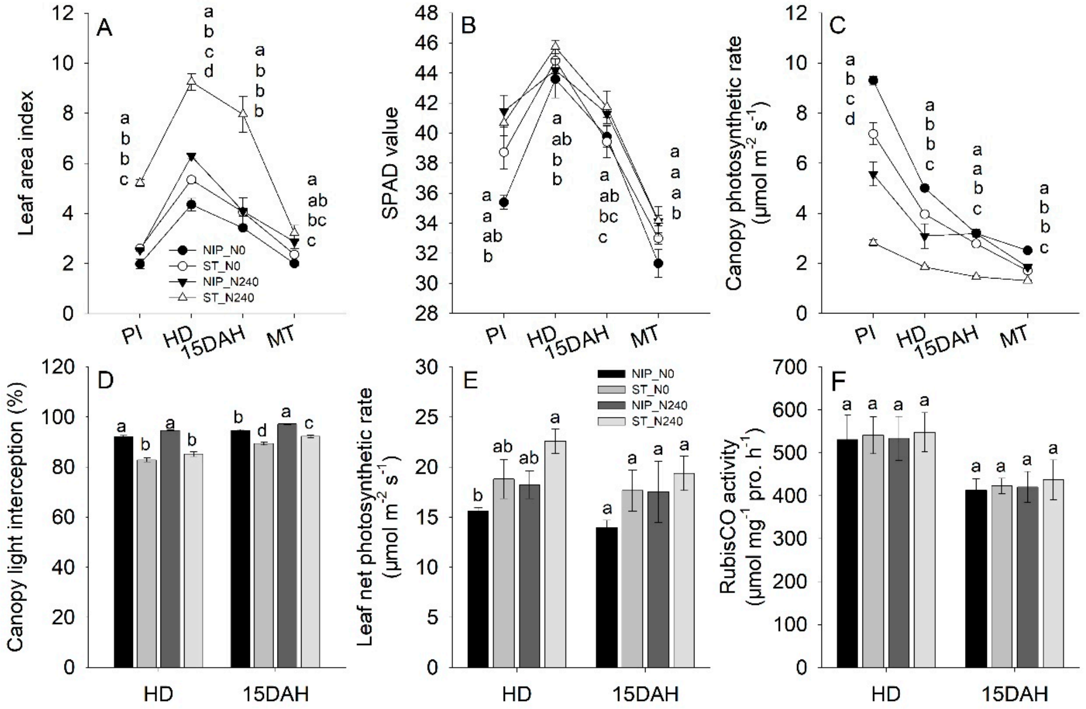

Figure S6. Rice photosynthetic capacity under different nitrogen treatments in 2024. NIP, Nipponbare; ST, ST-12. PI, jointing stage; HD, heading; DAH, day after heading; MT, maturity. Different letters indicate significant differences between treatments (p < 0.05)

(Fisher's least signiffcant difference (LSD) test).

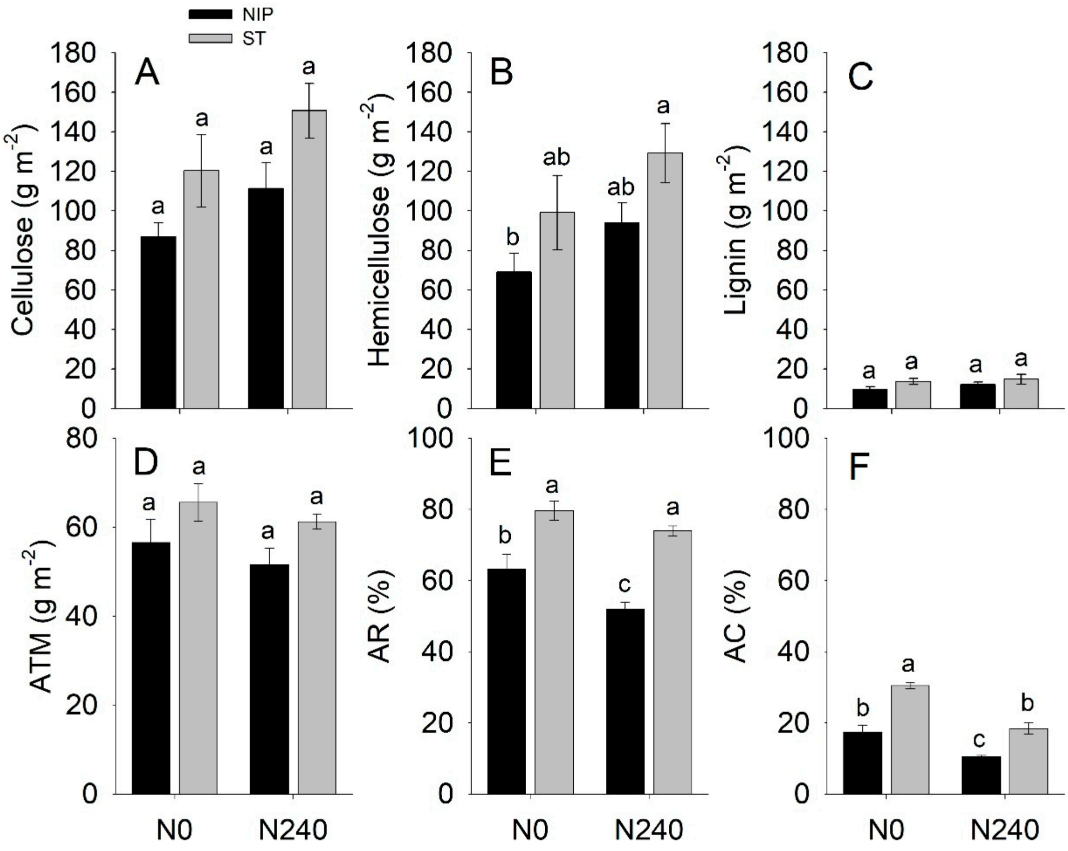

Figure S7. Rice carbohydrate accumulation and translocation under different nitrogen treatments in 2024. NIP, Nipponbare; ST, ST-12. Different letters indicate significant differences between treatments (p < 0.05) (Fisher's least signiffcant difference (LSD) test).

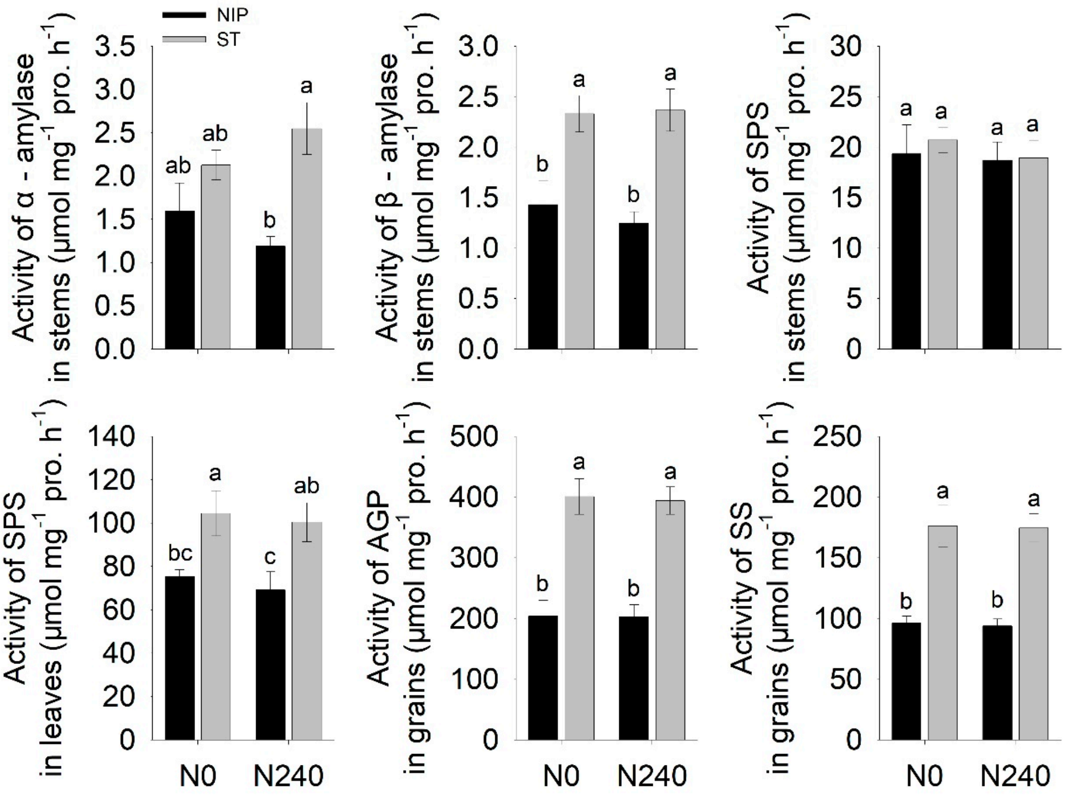

Figure S8. Carbon metabolism enzyme activities in rice plants under different nitrogen treatments in 2024. NIP, Nipponbare; ST, ST-12. Different letters indicate significant differences between treatments ($p < 0.05$) (Fisher's least signiffcant difference (LSD) test).

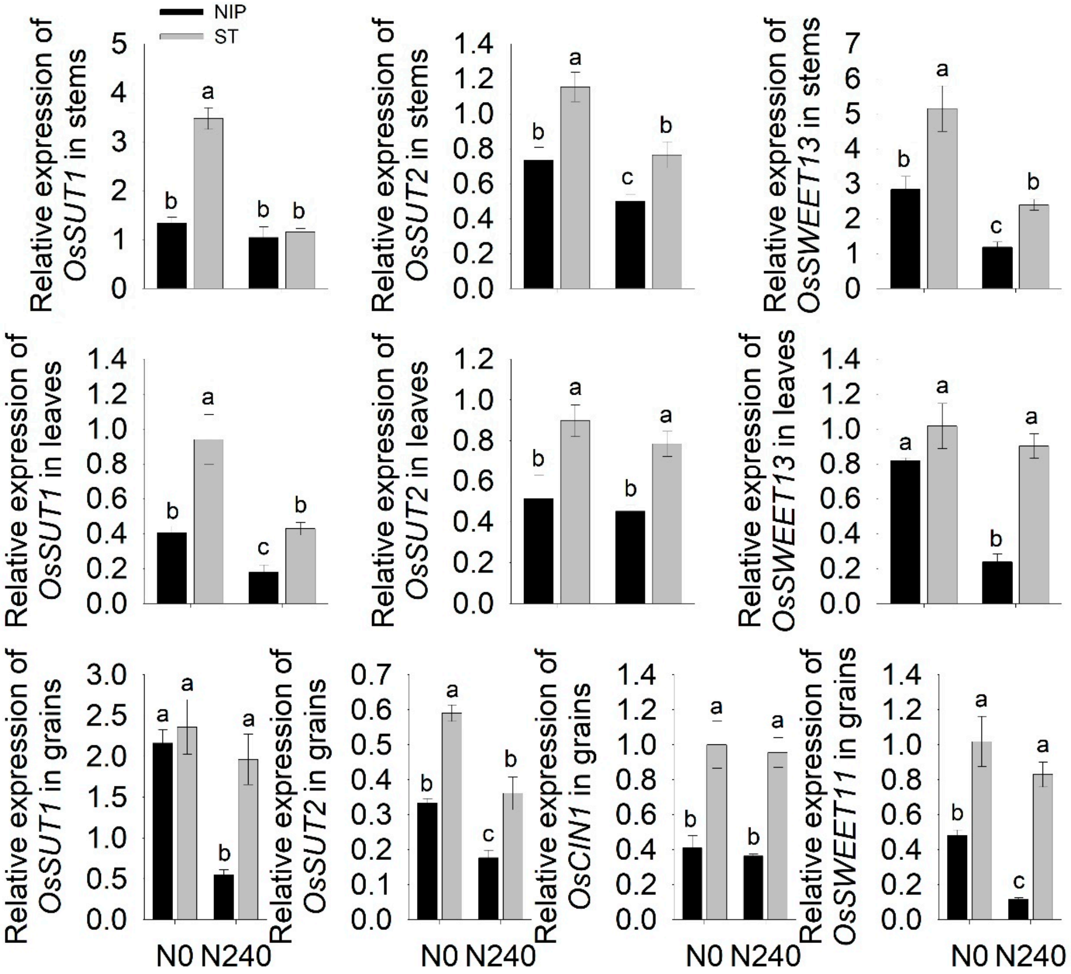

Figure S9. Relative expression levels of sugar transporter proteins in rice plants under different nitrogen treatments in 2024. NIP, Nipponbare; ST, ST-12. Different letters indicate significant differences between treatments ($p < 0.05$) (Fisher's least signiffcant difference (LSD) test).
